# Religious Affiliation and Flu Vaccination in Germany: Results of the German Ageing Survey

**DOI:** 10.3390/healthcare10102108

**Published:** 2022-10-21

**Authors:** Hamzah Shaheen, Hans-Helmut König, André Hajek

**Affiliations:** Department of Health Economics and Health Services Research, University Medical Center Hamburg-Eppendorf, Hamburg Center for Health Economics, 20246 Hamburg, Germany

**Keywords:** religious affiliation, religion, flu vaccination, influenza, church, preventive healthcare services

## Abstract

Our aim was to examine the association between religious affiliation and the likelihood of taking the flu vaccine. Cross-sectional data (year 2014 with *n* = 7172) were used from the nationally representative German Ageing Survey—covering community-dwelling individuals aged 40 years and over. Multiple logistic regressions showed that compared with individuals without a religious affiliation, individuals with certain religious affiliations had a lower likelihood of taking the flu vaccine. More precisely, the likelihood of taking a flu shot was significantly associated with belonging to the Roman Catholic Church (OR: 0.50, 95% CI: 0.44–0.57), the Protestant Church (OR: 0.68, 0.60–0.77), the Evangelic Free Church (OR: 0.54, 0.35–0.82) and other religious communities (OR: 0.25, 0.14–0.45). The results remained nearly the same when we restricted our analyses to individuals aged 60 years and over (according to existing recommendations for flu vaccination). The association between religious affiliation and the likelihood of taking the flu vaccine was moderated by thoughts about religion and deeds for religion. This knowledge could help to improve the immunization coverage by addressing individuals with certain religious affiliations.

## 1. Introduction

Influenza is a highly contagious viral infection caused by the influenza virus, which usually occurs during the winter months [1]. The challenge with this virus is that it mutates quite frequently resulting in new subtypes and strains [2].

The Robert Koch Institute (RKI) estimated in 2018/2019 a total of approximately 3.8 million influenza-associated illnesses, from which around 18,000 individuals were hospitalized in Germany [3]. Thus, this epidemic is a burden for healthcare systems every year [4]. The flu vaccination is effective in prevention. In Germany, only around 39% of above 65 years old were vaccinated with the influenza vaccine in 2019 which, in comparison to rates in countries such as in Chile (85%) or Korea (86%), is in great need of improvement [5]. The vaccination coverage in Germany between 2008 and 2011, for example, was much lower than for other diseases in the last 10 years such as Tetanus (71%), Diphtheria (82%) or Poliomyelitis (86%) [6]. The flu vaccination has an effectiveness between 40–60% and markedly reduces the risk for hospitalization, especially against influenza A(H1N1) and influenza B, among individuals in later life [7]. The influenza vaccine can also prevent deaths [8]. 

One of the most important groups for a flu shot are individuals aged ≥ 65 years since 90% of all influenza-associated hospitalizations and deaths occur in this age bracket [9]. That is why the WHO recommends an annual influenza vaccination for them. Despite the decreasing efficiency of the influenza vaccine with increasing age and frailty, it is still the most important prevention method for individuals in this age group [9]. 

Thus far, various studies have examined the determinants of the flu vaccination. For example, different studies found that age is positively associated with the willingness to take the influenza vaccine, such as the Garridos study [10]. Moreover, several studies determined that neither education nor income were significantly associated with the likelihood of taking a flu shot [11]. In addition, individuals who have a better understanding of the influenza virus and vaccine were more likely to get vaccinated [12]. Thus far, there is inconclusive evidence regarding the association between gender and the likelihood of taking a flu shot [13].

As shown above, there are a number of studies showing different (mainly classical sociodemographic) factors being associated with taking a flu shot. In contrast, addressing the gap in knowledge, there is limited knowledge regarding the association between religious factors (in terms of religious affiliation) and taking a flu shot. We are only aware of one study showing the different attitudes toward the flu vaccination of religious and non-religious groups. Therefore, our aim was to analyze the association between religious affiliation and flu vaccination among community-dwelling older adults (40 years and older) in Germany. Knowledge about an association between religious affiliation and the likelihood of taking a flu shot may assist in identifying certain groups at risk for comparably low vaccination rates. 

With regard to previous findings, Abramson’s study showed that individuals in Jerusalem who considered themselves religious had a lower rate of taking the influenza vaccine than non-religious individuals [14]. Another study conducted in Israel indicated that Christian and Muslim groups had a higher likelihood of getting vaccinated than Jewish groups [15]. Additionally, a study in Vanderbilt University Medical Center (VUMC) revealed that four out of the five most common reasons against vaccination were of a religious nature. The most common reason was that the “body is a temple or sacred” [16]. 

We observed that individuals with a religious affiliation have a lower likelihood of taking a flu shot compared with individuals with no religious affiliation, because they tend to see epidemics, pandemics and plagues as signs sent by God rather than from a scientific point of view, and therefore could have a higher level of doubt toward certain scientific opinions than non-religious individuals [17,18]. 

## 2. Materials and Methods

### 2.1. Sample

The data was provided by the German Ageing Survey (“Deutscher Alterssurvey”, DEAS), which is funded by the Federal Ministry for Family Affairs, Senior Citizens, Women and Youth in Germany. Baseline samples were drawn every 6 years starting from 1996. After every baseline sample there was a follow-up data collection (i.e., in wave 2, wave 3, and wave 5). Thus far, the survey has in total seven waves. The first wave took place in 1996, second wave 2002, third wave 2008, fourth wave 2011, fifth wave 2014, sixth wave 2017 and seventh wave 2020/2021. The DEAS study has a cohort-sequential design. The baseline samples included adults aged from 40 to 85 years. 

The response rate for the baseline samples decreased from 50.3% to 27.1% between 1996 and 2014, which is similar to, or even a little bit higher than some other surveys conducted in Germany [19]. Face-to-face interviews were conducted with a broad range of questions, for example, about general health or social support. Subsequently, individuals could fill out a drop-off questionnaire (including more sensitive questions, such as on life satisfaction). 

We focused on data collection in the year 2014 due to data availability. A total of 10,324 individuals participated in the DEAS interview in the year 2014. The drop-off questionnaire was filled out by 8039 individuals. In our analytical sample, n equaled 7172 individuals due to some missing values. Further details are provided by Klaus et al. [20]. 

Written informed consent was given by all participants prior to the study. Ethical approval for the DEAS study was not required because the criteria for need of an ethical statement were not fulfilled, such as the risk for respondents or use of invasive methods. 

### 2.2. Dependent Variable

The question was introduced as follows: “Doctors often recommend vaccinations and various types of health screening”. Flu vaccination was quantified as follows: “In the past years, did you regularly get a flu vaccination?” (no; yes). This assessment is in accordance with previous studies focusing on the use of preventive healthcare [21]. 

### 2.3. Independent Variables

Religious affiliation was quantified using a question as to which religion the participant belongs to (The Roman Catholic Church; The Protestant Church; An Evangelical Free Church; The Islamic religious community; Another religious community; No religious group). This is a common way to quantify the religious affiliation.

In regression analysis, the following sociodemographic covariates were included: Age, sex (men; women), marital status (Married, living together with spouse; Married, living separated from spouse; Divorced; Widowed; Single), monthly equivalence income (in Euro), education (ISCED classification (0–2: low; 3–4: medium; 5–6: high)), labor force participation (measured using three categories: employed; retired; other: not employed). 

The following health-related covariates were included in the regression analysis: Self-rated health and number of physical diseases. Self-rated health was quantified using a single item (1 = very good, 2 = good, 3 = average, 4 = bad, 5 = very bad), and the number of physical diseases was quantified by a count score of diseases (which ranged from 0 to 11: cardiac and circulatory disorders; bad circulation; Joint, bone, spinal and back problems; respiratory problems, asthma, shortness of breath; Stomach and intestinal problems; Cancer; Diabetes; Gall bladder, liver or kidney problems; Bladder problems; Eye problems, vision impairment; Ear problems, hearing problems). 

Additionally, we adjusted for the following variables: Thoughts regarding Religion (Ranging from 0 = Don’t think a lot about it to 5 = Think a lot about it) and Doing something for Religion (Ranging from 0 = Don’t do anything for it to 5 = Do a lot for it). The questions were introduced as follows: “In the following, we want to address your personal view on some issues and spheres of life. I would like to know, how much these issues bother you, so how much you think of them. In a second step I will ask you, how much you actively do for these issues and life spheres.”

### 2.4. Statistical Analysis

The sample characteristics were stratified by religious affiliation. One-way ANOVAs or Chi²-tests were conducted, as appropriate (*p*-values). Thereafter, multiple logistic regressions were carried out to analyze the association between religious affiliation and the likelihood of taking the flu vaccine. OR stands for odds ratio and CI for confidence interval. The level of statistical significance was set at 0.05. For the statistical analysis, Stata 17.0 (Stata Corp., College Station, TX, USA) was used. 

## 3. Results

### 3.1. Sample Characteristics

Sample characteristics (stratified by religious affiliation) are shown in Table 1. Average age was 64.3 years (SD: 11.6 years) and the age ranged from 40 to 95 years. In sum, 49.9% of the participants were female. Moreover, 7.8% of the individuals had a low education, 51.8% a medium education and 40.4% a high education. In total, 56.2% of the individuals did not take the flu shot regularly. 

Additionally, 64.5% of the Roman Catholics, 54.7% of the Protestants, 62.7% of the individuals of the Evangelic free church, 70.0% of the Muslims and 77.9% from another religious group did not get vaccinated. Moreover, 50.7% individuals with no religious affiliation did not take the flu shot. Further details are provided in Table 1. According to a Chi²-test, the association of interest (i.e., between religious affiliation and flu vaccination) is significant (*p* < 0.001). Moreover, the religious affiliation was significantly associated with sex (*p* < 0.001), labor force status (*p* < 0.001), marital status (*p* < 0.001), educational level (*p* < 0.001), and self-rated health (*p* < 0.05); whereas it was not significantly associated with the total number of physical conditions (*p* = 0.07).

According to a one-way ANOVA, there were significant differences in age (*p* < 0.001) and income (*p* < 0.001) between the different religious affiliations. More precisely (according to Bonferroni multiple-comparison tests), individuals belonging to the Roman Catholic Church differ in terms of income from individuals belonging to the Islamic religious community (*p* < 0.001). Individuals belonging to the Roman Catholic Church differ in terms of income from individuals belonging to the group of “another religious community” (*p* < 0.01). Individuals belonging to the Protestant Church differ in terms of income from individuals belonging to the Islamic religious community (*p* < 0.001). Individuals belonging to the Protestant Church differ in terms of income from individuals belonging to the group of “another religious community” (*p* < 0.05). Individuals belonging to the Evangelical Free Church differ in terms of income from individuals belonging to the Islamic religious community (*p* < 0.01). Individuals belonging to the Islamic religious community differ in terms of income from individuals belonging to no religious group (*p* < 0.001). 

Moreover, individuals belonging to the Roman Catholic Church differ in terms of age from individuals belonging to the Protestant Church (*p* < 0.001). Individuals belonging to the Roman Catholic Church differ in terms of age from individuals belonging to the Islamic religious community (*p* < 0.001). Individuals belonging to the Roman Catholic Church differ in terms of age from individuals belonging to no religious group (*p* < 0.05). Individuals belonging to the Protestant Church differ in terms of age from individuals belonging to the Islamic religious community (*p* < 0.001). Individuals belonging to the Protestant Church differ in terms of age from individuals belonging to no religious group (*p* < 0.001). Individuals belonging to the Evangelical Free Church differ in terms of age from individuals belonging to the Islamic religious community (*p* < 0.001). Individuals belonging to the Islamic religious community differ in terms of age from individuals belonging to the group of “another religious community” (*p* < 0.01). Individuals belonging to the Islamic religious community differ in terms of age from individuals belonging to no religious group (*p* < 0.001). 

### 3.2. Regression Analysis

The findings of the multiple logistic regressions are shown in Table 2 (unadjusted regressions are given in Supplementary Table S1). Adjusted ORs are presented (95% CI in parentheses). It was adjusted for sex, age, level of education, marital status, labor force status, monthly income, self-rated health, and the total number of physical diseases.

Regression analysis showed that compared with individuals without a religious affiliation, individuals with a certain religious affiliation had a lower likelihood of taking the flu vaccine; for example, it was significantly associated with belonging to the Roman Catholic Church, belonging to the Protestant Church, belonging to the Evangelic Free Church and belonging to other religious communities; whereas it was not significantly associated with belonging to the Islamic religious community.

Additionally, the uptake of the flu vaccine was positively associated with an increase in age (OR: 1.05, 1.04–1.06), being retired (compared to being employed, OR: 1.45, 1.22–1.72), having a medium (compared to low education, OR: 1.26, 1.01–1.56) or high education (OR: 1.26, 1.00–1.58), lower income (OR: 0.999942, 0.9998994–0.9999847), worse self-rated health (OR: 1.24, 1.16–1.33) and a higher number of chronic diseases (OR: 1.08, 1.05–1.12). 

In additional analysis, we restricted our sample to individuals aged 60 years and above (in accordance with the Standing Committee on Vaccination (STIKO) recommendations for flu vaccination). The association between religious affiliation and the likelihood of taking a flu shot remained similar in terms of significance and effect size in this age group.

Furthermore, in further analysis, we tested whether the association between religious affiliation and the likelihood of taking a flu shot was moderated by “Thoughts regarding Religion” (by adding interaction terms: religious affiliation x thoughts regarding religion). There was a significant interaction between “Thoughts regarding Religion” and belonging to a religious affiliation. More precisely, the interaction terms for religious affiliation (i.e., belonging to the Roman Catholic (*p* < 0.001) or Protestant Church; compared with individuals without a religious affiliation (*p* < 0.01)); and thoughts regarding affiliation achieved statistical significance.

In further additional analysis, we tested whether the association between religious affiliation and the likelihood of taking a flu shot was moderated by “Doing something for Religion” (by adding an interaction term: religious affiliation x actions regarding religion). There was a significant interaction between actions regarding religion and belonging to a religious affiliation (i.e., belonging to the Roman Catholic (*p* < 0.001) or Protestant Church (*p* < 0.01); compared with individuals without a religious affiliation; please see Supplementary Tables S2 and S3). 

This additional analysis showed that people from the Roman Catholic or Protestant Church, who have less thoughts regarding Religion and do less for Religion, are less likely to get vaccinated than non-religious people.

## 4. Discussion

### 4.1. Main Findings

Based on a large representative sample, the aim of this study was to analyze the association between religious affiliation and flu vaccination among community-dwelling older adults (40 years and older) in Germany. Our results showed that individuals with certain religious affiliation had a lower likelihood of taking the flu vaccine. More precisely, the likelihood of taking a flu shot was significantly negatively associated with belonging to the Roman Catholic Church, Protestant Church, Evangelic Free Church and belonging to other religious communities. 

### 4.2. Relation to Previous Research and Possible Explanations 

We are only aware of one study showing the difference in religious and non-religious groups in terms of flu vaccination. Thus, due to the very restricted knowledge, it is difficult to compare our study with prior research [22]. 

There could be several reasons for the associations found in our study. One possible explanation could be the level of distrust of some with religious affiliations to science or scientific advances. For some institutions, science is a threat to their beliefs, and they fear that Atheists are determining what science looks like. 

It may be the case that these groups do not perceive science as a contradiction to religion, but rather as a threat to their social identify. An underlying reason may be their perceived association between science and atheism. The Evangelicals in the US, for instance, supported the Anti-Vaccination movement during the COVID-19 pandemic, because they felt that the health guidelines restricted their religious freedom and freedom of expression [23]. Furthermore, some religious people (e.g., White Evangelicals) might think that if one takes the vaccine, it might interfere with their faith and trust in God, because they may think that “God is the only Healer” [23]. As aforementioned, the epidemics or the recent COVID-19 pandemic may be seen as trial or sign by God to test their faith. To stay loyal to their faith, the hesitancy to take vaccines could be increased in such individuals. 

We did not find a statistically significant association between religious affiliation and a decreased likelihood of taking the flu vaccine for the Islamic religious community. This could be due to the reason that in Islam, science and scientific advances were already important in the beginning of its history. That time was called “The Golden Age” in which the Muslims studied and searched for knowledge. They upheld their religious doctrines but were involved in scientific research as well [24]. However, it should be emphasized that it could be explained by the smaller sample size for the Islamic Religious Community (*n* = 32). Thus, the missing association may also be explained by a lack of statistical power. Our findings should be therefore interpreted with great caution. Future research with a higher number of individuals with an Islamic affiliation is urgently required to confirm our findings. On the other hand, the religion could be used by individuals with personal reluctance against vaccines as an excuse not to vaccinate themselves. The policy of religious exemption in the US, for example, is used by “Anti-Vaxxers”, because it is hard to prove if the reason is of s religious nature or because of some personal objection [25]. In all major religions the use of vaccines is allowed, and the most common objections can be invalidated theologically [26]. Only some minor denominations (e.g., Dutch Reformed Congregations, Church of the First Born, Faith Assembly, Nation of Islam) have a religious objection to vaccines [27]. In some cases, the religion affiliation may also be misused because of social and political issues. One example is Pakistan, where a Polio resurgence occurred due to some statements of Muslim fundamentalists, spreading rumors about Western conspiracies [28]. Thus, it may be the case that for most people the objection against vaccinations is more traditional or social and not because of any theological aspect. On the contrary, the major religions also recommend vaccinations to save lives [27]. 

From a public health perspective, it may be beneficial to educate individuals in a variety of ways. This can potentially lead to a behavior change toward vaccination. This could not only contribute to the health of individuals, but also others. A study conducted by Kuru showed that some religious beliefs that conflict with vaccinations, tend to negatively affect the intention to encourage others to vaccinate [29]. This could be done, among other things, on a governmental level by informational campaigns about religion and vaccination, by general practitioners or by religious leaders. 

During the COVID-19 pandemic, public mistrust in governments could be partly explained by misinformation spread on social media platforms [30]. One way to increase flu vaccination rates could be via promoting flu vaccination by celebrities and influencers on such social media platforms.

It has been shown that there is an association between Religion and Belief in Science in all major religions. This connection, which can be found in the holy scriptures as well, can be used to address the aim to have an increased vaccination rate [31]. One potential way of doing this is to form a committee of the religious leaders in Germany and representatives of the healthcare system. The religious leaders can then later motivate the believers of their religion to vaccinate themselves. The general practitioner can increase the vaccination rate as well by communicating the importance of health and care of the human body (which is also part of every major religion) to the patient [32].

We assume that a key reason behind the reluctance toward vaccinations is not because of the Religion itself, but because of religious ignorance or personal reasons (e.g., distrust of vaccines due to misinformation on social media, mistrust in political authorities or the pharmaceutical industry). However, future research is urgently required to test our assumptions.

### 4.3. Strengths and Limitations

A major strength of our study is that data were used from a large, nationally representative sample of individuals aged 40 years and above residing in private households. This allows our results to be applied for the general community-dwelling population in the second half of life. This study is also the first study to examine the association between religious affiliation and flu vaccination in Germany. Additionally, we distinguished between several religious groups in our study. The key questions in the study were commonly understandable and easy to answer. Nevertheless, more detailed questions about the reasons for the vaccination or reluctance of vaccinations could be asked. Thus, future research in this area is required. 

Our study also has some limitations. It may be difficult to generalize our findings to, e.g., individuals in very late life or individuals with low education because the participation in the DEAS study to a certain extend depends on characteristics like education, or age group. This sample selection bias, however, is rather small [20]. Moreover, the distribution of key sociodemographic factors (e.g., family situation, labor force participation, or educational level) is very close compared with the distribution within the German population [33]. The causality between the religious affiliation and likelihood of taking a flu shot is also not fully clear. It could be that the likelihood of taking a flu shot could lead to a change in religious affiliation. However, it should be noted that this directionality seems rather unlikely. It should be noted that the count score for the number of physical diseases has some shortcomings (e.g., distinguishing between circulatory disorder and bad circulation).

## 5. Conclusions

(1) Our study findings showed that there is a clear link between having a religious affiliation (i.e., belonging to the Roman Catholic Church, the Protestant Church, the Evangelic Free Church and other religious communities; compared with individuals without a religious affiliation) and a decreased likelihood of taking the flu vaccine—based on data from a large nationally representative sample and after adjusting for various covariates in the regression analysis.
(2) We think that this could help to improve the vaccination coverage by addressing individuals with certain religious affiliations (i.e., individuals with a Roman Catholic or Evangelic background).
(3) This knowledge is important, among other information, for policy makers, public health experts and physicians.

## Figures and Tables

**Table 1:** Sample characteristics (stratified by religious affiliation).

	The Roman Catholic Church	The Protestant Church (Not Including Free Churches)	An Evangelical Free Church	The Islamic Religious Community	Another Religious Community	No Religious Group	*p*-Value
	*n* = 2114 (26.8%)	*n* = 2551 (32.3%)	*n* = 120 (1.5%)	*n* = 32 (0.4%)	*n* = 80 (1.0%)	*n* = 2992 (37.9%)	
	Mean (SD)/*n* (%)	Mean (SD)/*n* (%)	Mean (SD)/*n* (%)	Mean (SD)/*n* (%)	Mean (SD)/*n* (%)	Mean (SD)/*n* (%)	
Participation: flu vaccination							*p* < 0.001
Yes	730 (35.5%)	1125 (45.3%)	44 (37.3%)	9 (30.0%)	17 (22.1%)	1449 (49.3%)	
No	1327 (64.5%)	1360 (54.7%)	74 (62.7%)	21 (70.0%)	60 (77.9%)	1492 (50.7%)	
Age	64.4 (11.1)	66.1 (11.4)	63.9 (12.2)	54.9 (10.5)	63.3 (12.0)	63.4 (10.9)	*p* < 0.001
Sex							*p* < 0.001
Male	993 (47.0%)	1151 (45.1%)	46 (38.3%)	20 (62.5%)	49 (61.3%)	1606 (53.7%)	
Female	1121 (53.0%)	1400 (54.9%)	74 (61.7%)	12 (37.5%)	31 (38.8%)	1386 (46.3%)	
Labour force status							*p* < 0.001
Working	820 (38.8%)	809 (31.7%)	45 (37.5%)	12 (37.5%)	23 (28.7%)	1155 (38.6%)	
Retired	1090 (51.6%)	1525 (59.8%)	65 (54.2%)	12 (37.5%)	48 (60.0%)	1576 (52.7%)	
Other: not employed	203 (9.6%)	215 (8.4%)	10 (8.3%)	8 (25.0%)	9 (11.3%)	260 (8.7%)	
Marital status							*p* < 0.001
Married, living together with spouse	1568 (74.2%)	1793 (70.4%)	80 (66.7%)	24 (75.0%)	54 (67.5%)	1994 (66.9%)	
Married, living separated from spouse	28 (1.3%)	44 (1.7%)	1 (0.8%)	0 (0.0%)	1 (1.3%)	53 (1.8%)	
Divorced	149 (7.1%)	226 (8.9%)	14 (11.7%)	7 (21.9%)	12 (15.0%)	376 (12.6%)	
Widowed	228 (10.8%)	335 (13.2%)	15 (12.5%)	1 (3.1%)	8 (10.0%)	305 (10.2%)	
Single	140 (6.6%)	148 (5.8%)	10 (8.3%)	0 (0.0%)	5 (6.3%)	254 (8.5%)	
Monthly equivalence income (in EUR)	2027.6 (1490.5)	1933.5 (1171.1)	1879.7 (1584.8)	797.2 (324.4)	1440.6 (846.9)	1912.4 (1460.4)	*p* < 0.001
Level of education (ISCED-classification)							*p* < 0.001
Low (ISCED 0–2)	211 (10.0%)	179 (7.0%)	17 (14.2%)	19 (59.4%)	11 (13.8%)	85 (2.8%)	
Medium (ISCED 3–4)	1152 (54.5%)	1334 (52.3%)	67 (55.8%)	10 (31.3%)	40 (50.0%)	1459 (48.8%)	
High (ISCED 5–6)	750 (35.5%)	1037 (40.7%)	36 (30.0%)	3 (9.4%)	29 (36.3%)	1448 (48.4%)	
Self rated state of health							*p* < 0.05
Very good	172 (8.1%)	197 (7.7%)	7 (5.8%)	1 (3.1%)	2 (2.5%)	256 (8.6%)	
Good	1009 (47.8%)	1155 (45.3%)	54 (45.0%)	14 (43.8%)	37 (46.3%)	1342 (44.9%)	
Average	736 (34.8%)	942 (37.0%)	47 (39.2%)	10 (31.3%)	32 (40.0%)	1053 (35.2%)	
Bad	172 (8.1%)	213 (8.4%)	10 (8.3%)	4 (12.5%)	6 (7.5%)	269 (9.0%)	
Very bad	24 (1.1%)	41 (1.6%)	2 (1.7%)	3 (9.4%)	3 (3.8%)	68 (2.3%)	
Total number of physical conditions							*p* = 0.07
0	220 (10.6%)	272 (10.9%)	9 (7.8%)	5 (15.6%)	7 (8.8%)	360 (12.2%)	
1	443 (21.3%)	503 (20.1%)	18 (15.5%)	8 (25.0%)	16 (20.0%)	640 (21.7%)	
2	466 (22.4%)	572 (22.9%)	33 (28.4%)	7 (21.9%)	12 (15.0%)	643 (21.8%)	
3	361 (17.3%)	418 (16.7%)	18 (15.5%)	1 (3.1%)	11 (13.8%)	519 (17.6%)	
4 or more	593 (28.5%)	737 (29.5%)	38 (32.8%)	11 (34.4%)	34 (42.5%)	786 (26.7%)	

Notes: One-way ANOVAs or Chi^2^-tests were conducted, as appropriate (*p*-values).

**Table 2:** Association between religious affiliation and likelihood of taking a flu shot (0 = no; 1 = yes). Results of multiple logistic regressions adjusted for potential confounders described in the notes.

Independent Variables	Likelihood of Taking a Flu Shot
	OR (95% CI)
Religious affiliation: The Roman Catholic Church (Ref.: no religious affiliation)	0.50 *** (0.44–0.57)
The Protestant Church (not including free churches)	0.68 *** (0.60–0.77)
An Evangelical Free Church	0.54 ** (0.35–0.82)
The Islamic religious community	0.52 (0.22–1.25)
Another religious community	0.25 *** (0.14–0.45)
Potential confounders	✓
Pseudo R²	0.11
Observations	7172

Notes: *** *p* < 0.001, ** *p* < 0.01; Potential confounders include sex, age, level of education, marital status, labor force status, monthly income, self-rated health, and the total number of physical diseases.

## Data Availability

The data used in this study are third-party data. The anonymized data sets of the DEAS (1996, 2002, 2008, 2011, 2014, 2017 and 2020) are available for secondary analysis. The data have been made available to scientists at universities and research institutes exclusively for scientific purposes. The use of data is subject to written data protection agreements. Microdata of the German Ageing Survey (DEAS) are available free of charge to scientific researchers for non-profitable purposes. The FDZ-DZA provides access and support to scholars interested in using DEAS for their research. However, for reasons of data protection, signing a data distribution contract is required before data can be obtained. For further information on the data distribution contract, please see https://www.dza.de/en/research/fdz/access-to-data/formular-deas-en-english (accessed on 1 September 2022).

## Supplementary Materials

The following are available online at https://www.mdpi.com/article/10.3390/healthcare10102108/s1, Supplementary Table S1: Association between religious affiliation and likelihood of taking a flu shot (0 = no; 1 = yes). Results of binary logistic regressions (unadjusted). Supplementary Table S2: Association between religious affiliation and likelihood of taking a flu shot (0 = no; 1 = yes). Results of multiple logistic regressions (with interaction terms: religious affiliation × Thoughts regarding Religion). Supplementary Table S3: Association between religious affiliation and likelihood of taking a flu shot (0 = no; 1 = yes). Results of multiple logistic regressions (with interaction terms: religious affiliation x Doing something regarding Religion).
