# Supplementary material for: Religious Affiliation and Flu Vaccination in Germany: Results of the German Ageing Survey
Source: Healthcare (Basel). 2022 Oct 21;10(10):2108. doi: 10.3390/healthcare10102108 (PMC9602020; doi:10.3390/healthcare10102108)
Supplement: Supplementary file 1 [file healthcare-10-02108-s001.zip › healthcare-1932093-supplementary.pdf]

Supplementary Table S1. Association between religious affiliation and likelihood of taking a flu shot (0 = no; 1 = yes). Results of binary logistic regressions (unadjusted)

Independent variables	Likelihood of taking a flu shot
Religious affiliation: - The Roman Catholic Church (Ref.: no religious affiliation)	0.57*** (0.50-0.64)
- The Protestant Church (not including free churches)	0.85** (0.77-0.95)
- An Evangelical Free Church	0.61* (0.42-0.90)
- The Islamic religious community	0.44* (0.20-0.97)
- Another religious community	0.29*** (0.17-0.50)
Pseudo R^2	0.01
Observations	7,708

Notes: Odds Ratios are reported; 95% CI in parentheses; *** p<0.001, ** p<0.01, * p<0.05.

Supplementary Table S2. Association between religious affiliation and likelihood of taking a flu shot (0 = no; 1 = yes). Results of multiple logistic regressions (with interaction terms: religious affiliation x Thoughts regarding Religion).

Independent variables	Likelihood of taking a flu shot
Religious affiliation: - The Roman Catholic Church (Ref.: no religious affiliation)	0.38*** (0.30-0.47)
- The Protestant Church (not including free churches)	0.61*** (0.51-0.74)
- An Evangelical Free Church	0.79 (0.30-2.07)
- The Islamic religious community	0.53 (0.08-3.34)
- Another religious community	1.13 (0.32-4.01)
Thoughts regarding Religion	0.86*** (0.80-0.91)
Interaction terms: - The Roman Catholic Church (Ref.: no religious affiliation) x Thoughts regarding Religion	1.24*** (1.13-1.35)
- The Protestant Church (not including free churches) x Thoughts regarding Religion	1.16** (1.06-1.26)
- An Evangelical Free Church x Thoughts regarding Religion	1.01 (0.79-1.31)
- The Islamic religious community x Thoughts regarding Religion	1.08 (0.65-1.80)
- Another religious community x Thoughts regarding Religion	0.74+ (0.53-1.04)
Potential confounders	✓
Pseudo R^2	0.11
Observations	7,162

Notes: Odds Ratios are reported; 95% CI in parentheses; *** p<0.001, ** p<0.01, + p<0.10; Potential confounders include sex, age, level of education, marital status, labour force status, monthly income, self-rated health, and the total number of physical diseases

Supplementary Table S3. Association between religious affiliation and likelihood of taking a flu shot (0 = no; 1 = yes). Results of multiple logistic regressions (with interaction terms: religious affiliation x Doing something regarding Religion).

Independent variables	Likelihood of taking a flu shot
Religious affiliation: - The Roman Catholic Church (Ref.: no religious affiliation)	0.41*** (0.34-0.50)
- The Protestant Church (not including free churches)	0.60*** (0.51-0.71)
- An Evangelical Free Church	0.79 (0.34-1.85)
- The Islamic religious community	0.91 (0.17-4.97)
- Another religious community	0.86 (0.28-2.64)
Doing something regarding Religion	0.86** (0.80-0.94)
Interaction terms: - The Roman Catholic Church (Ref.: no religious affiliation) x Doing something regarding Religion	1.21*** (1.09-1.35)
- The Protestant Church (not including free churches) x Doing something regarding Religion	1.18** (1.07-1.31)
- An Evangelical Free Church x Doing something regarding Religion	1.00 (0.78-1.28)
- The Islamic religious community x Doing something regarding Religion	0.91 (0.55-1.49)
- Another religious community x Doing something regarding Religion	0.78 (0.56-1.07)
Potential confounders	✓
Pseudo R^2	0.11
Observations	7,153

Notes: Odds Ratios are reported; 95% CI in parentheses; *** p<0.001, ** p<0.01; Potential confounders include sex, age, level of education, marital status, labour force status, monthly income, self-rated health, and the total number of physical diseases
